# Regulatory landscapes of five mental health professions in Europe - a descriptive study based on the insights from the European Commission database

**DOI:** 10.1186/s12913-026-14469-3

**Published:** 2026-04-03

**Authors:** Nina Kilkku, Clare Lewis, Thomas Kearns, Michael Shannon

**Affiliations:** 1 VID Specialized University, P.O. Box 184, NO-0319 Vinderen, Oslo, Norway; 2 Royal College of Surgeons in Ireland, Dublin, Ireland

**Keywords:** Profession, Transformation, Mental health services, Regulation, Workforce, Descriptive study

## Abstract

**Background:**

In European countries, the reform and transformation of mental health services towards a community-based service system is at different stages. It is evident that professionals working in this field have a pivotal role to play in this development. However, there is a paucity of information regarding these professionals, which makes it difficult to support and monitor the change from the perspective of the workforce.

**Methods:**

The aim of this study was to fulfil this knowledge gap by describing the currently available information on the regulation, the qualification and education levels, duration of education, and description of activities of the five mental health professions in Europe. The included professions were psychiatrists, psychologists, psychiatric and mental health nurses, social workers, and occupational therapists. Data was collected from the European Commission’s open database of regulated professions at the end of 2024 and analyzed using descriptive analysis methods.

**Results:**

The results of this study demonstrate significant diversity across Europe among the five professions. This includes differences in regulatory practices and education, as well as differences in the description of activities for each profession.

**Conclusions:**

As a conclusion development of regulative practices is suggested as one solution to ensure the competence of the workforce and further the quality of mental health care. The results of this descriptive study highlight the need for further research into professional competency equivalence and professional development requirements, in order to support the transformation of mental health services in Europe.

## Background

In several European countries and globally, the transformation of mental health services based on the human rights-based approach [1, 2] with the reform from institutionalized care to community-based services, has been ongoing for several decades [3, 4]. This transformation has many aims, such as early identification and care in different mental health conditions, thereby improving prognosis [5, 6] and limiting the costs of care [7, 8]. As different historical, political, economic, cultural and geographical factors are influencing this development [3, 9], countries are at very different stages in this process, although many strategies, policies and documents are supporting and emphasizing this reform [1, 10].

Mental health care is based on person-centered encounters, human interaction [11] and therefore transformation is highly dependent on the well-trained and competent workforce in different levels of service system [4, 12–14]. However, the information on the mental health workforce is limited [10]. Whilst the Mental Health Atlas [15] does provide information regarding the number of mental health professionals, there is a paucity of knowledge concerning other information on these professions in European countries. From the perspective of mental health service transformation, interesting factors also include changes in the competence and scope of practice of different professionals as service provision shifts from institutions to primary health care and community-based services [16]. This absence of knowledge presents a challenge to support and monitor service development from the perspective of the workforce.

Regulation is a process that can provide information about professional capacity and capability of the workforce, although concerns have been raised about other aspects of regulations [17]. In social and health care services in Europe, the focus of regulatory practice is often on patient and public safety [18, 19], informed by risk, clinical incidents and the public’s declining trust in healthcare and professionals [20]. The EU Commission defines a regulated profession as “*a profession where access and exercise is subject to the possession of a specific professional qualification”* [21]. For some professions this means that the title of the profession is protected by law, which provides a level of assurance that individuals are appropriately trained and qualified in that profession, are registered (the recognition of qualifications to practice in the role), and are competent (regulation of the profession to practice in the role and use the title), to meet safe and professional standards of practice [21, 22]. However, differences exist between the countries on regulative practices in Europe [23], although in EU member states, European Directives cover the minimum education and training requirements for Doctor of Medicine, specialized doctors, nurses, dental practitioners, veterinary surgeons, midwives, pharmacists and architects [21, 24]. Other key professions in mental health, such as psychologists, social workers, and occupational therapists, are not covered by these directives.

The presented study focuses on five mental health professions: psychiatrists, psychiatric and mental health nurses, psychologists, social workers, and occupational therapists. These professions were considered to have a significant role in the transformation of various levels of service system. The aim of the study was to describe the currently available information on the regulation, the qualification and education levels, duration of education, and description of activities (the description of activities is the definition used in the database, often as a synonym with scope of practice) of these professions in Europe. The objectives of the study were to:

identify the types of regulation applied to psychiatrists, psychologists, psychiatric and mental health nurses, social workers, and occupational therapists across Europe;

describe variations in qualification levels and duration of education and training for these professions; and

examine the reported descriptions of activities across professions and countries.

## Methods

This descriptive study provides cross-sectional knowledge based on a single dataset and data collection at a specific point at the end of 2024. The data was collected using the data fields included in the EU Commission’s Regulated Professions Database [25]. This open-source database contains information on regulated professions, statistics on migrating professionals, contact points and competent authorities, as provided by 32 countries in Europe, including EU Member States, EEA countries, the UK and Switzerland. Each country is responsible for providing this information on the database. In the present study the data was collected using the information from the sections “Generic Names of the Professions” and “Regulated Professions by Country, with Competent Authorities” in the database. The first one indicates the number of the regulated professions, for example for occupational therapists 29 entries, and the latter gives more precise information on the name of the regulated profession, country, region and recognition under Directive 2005/36/EC.

The database recognizes professional titles such as social worker, occupational therapist, and psychologist, but the filters do not recognize the professional titles of doctors and nurses such as psychiatrist or psychiatric/mental health nurse. These were searched for using the generic term and the results were screened manually to recognize the specialization. Greek and Russian language data were excluded.

As no similar study had been conducted in Europe before, the aim was set at the descriptive level. This decision was further supported by observations of the quality of the data during the collection phase.

To analyze the data, STATA version 18 was employed to examine descriptive statistics. This involved comparing averages, such as means and medians, to report the central tendency of the variables. Data was analyzed with general comparisons on regulatory practices including titles, the level of qualification and education, duration of training and education and description of activities. Types of regulation were analyzed according to descriptions provided by European Commission [26] (Table 1), additionally some countries were using also “regulated under national registration”.

**Table 1:** Types of regulations with descriptions [26]

Type of Regulation	Description
Reserves of Activities	Professions where certain activities are reserved for the holders of a specific professional qualification.
Reserves of Activities and a Protected Title	Professions where there are both reserves of activities and a protected title.
Protected Title (without reserves of activities)	Professions where only the title is protected.

The qualification and education levels are described in the database as (i) DSE diploma post-secondary education, (ii) PS3-diploma in secondary level education, 3–4 years, (iii) PS4-diploma of post-secondary level, 4 years, and (iv) PSM diploma of post-secondary level, more than 4 years. These same descriptions were used to analyze these levels in data. Besides these four levels, a diploma-post secondary level, more than 4 years and SEC-certificate attesting to the completion of a secondary level course was found in data.

A qualitative descriptive approach was applied in the analysis of the descriptions of activities. The textual descriptions provided in the database were systematically reviewed for each profession and the recurrent, common terms were identified. As the descriptions in the database were limited in length and content, this process did not constitute a formal qualitative content analysis but was undertaken to enable structured comparisons. Data for each profession was analyzed separately to account for their distinct professional roles. The results are reported according to the common terms that emerged from the data.

## Results

The results are first reported with the summary tables on the types of regulation, the levels of qualification and education, and duration of education as reported by the countries (Tables 2, 3 and 4). This information is followed by detailed descriptions of each profession, including the description of activities.

### Types of regulation

The results on the type of regulation show that the most common type of regulation for psychiatrists, psychiatric and mental health nurses and occupational therapists was a ‘protected title with reserves of activities’ while regulation for social workers was most often a ‘protected title without reserves of activities’ (Table 2). Differences occurred in the case of the profession of psychologist, which was reported as ‘a protected title without reserves of activities’, but which was also regulated by national legislation in some countries.

The number of reporting countries was generally low, except for reports on psychiatrists and psychologists. This reflects the low number of countries that regulate the professions of psychiatric or mental health nurses, occupational therapists, and social workers.

**Table 2:** Types of regulation and number of countries reporting across the five professions

Profession	Number of countries reporting a protected title with reserves of activities	Number of countries reporting protected title without reserves of activities	Number of countries reporting reserves of activities	Regulated under national legislation
Psychiatrist	32	-	-	-
Psychiatric/Mental Health Nurse	12	-	-	-
Psychologist	9	10	3	6
Occupational Therapist	6	2	4	-
Social Worker	1	6	2	-

### Qualification and levels of education

The results concerning the qualification and education levels for each profession are reported in Table 3, in which the numbers in the columns indicate the number of reporting countries. All countries reported the qualification and education level to psychiatrists as “PSM diploma of post-secondary level, more than 4 years”. Similarly, almost all countries reported psychologists on this level or as “Diploma-Post secondary level, more than 4 years”, but with the other professions there was large variations between countries and again, the number of reporting countries was low.

**Table 3:** Qualification and educational levels reported by countries

Qualification and educational levels						
Qualification and Education levels/ number of countries reporting	DSE diploma post-secondary education	PS3-Diploma in secondary level education 3–4 years	PS4- diploma of post-secondary level 4 years	PSM diploma of post-secondary level, more than 4 years	Diploma-Post secondary level, more than 4 years	SEC-Certificate attesting to the completion of a secondary level course
Psychiatrists		***-***	***-***	*32*	***-***	***-***
Psychiatric/ Mental Health Nurses	2	3	3	3	**-**	1
Psychologists		***-***	***-***	27	2	***-***
Occupational therapists	2	9	***-***	1	***-***	*-*
Social workers	1	4	1	2	***-***	*-*

### Duration of education and training

Durations of education and training are based on the number of reporting countries, which vary for each profession (Table 4). Therefore, average durations may be higher or lower in countries that have not provided data.

**Table 4:** Average duration of education and training and minimum and maximum durations

Profession	Average duration of education and training in years	Minimum duration of education and training in years	Maximum duration of education and training in years
Psychiatrist	5.17	2	10
Psychiatric/Mental Health Nurse	2	0.5	4
Psychologist	5.19	3	10
Occupational Therapist	3.5	2	5
Social Worker	4.25	3	5

Figures show substantial differences between the minimum and maximum duration which could reflect different reporting practices, but also differences in the educational structure. These differences are described in more detailed in the following results for each profession.

## Psychiatrists

### Regulatory practices and duration of education and training

All 32 countries regulate and recognize the role of psychiatrist as a protected title with reserves of activities (Table 1). There were several terms used to describe the profession: adult psychiatry, child psychiatry, child and adolescent psychiatry, neuropsychiatry, and forensic psychiatry.

**Fig. 1:**
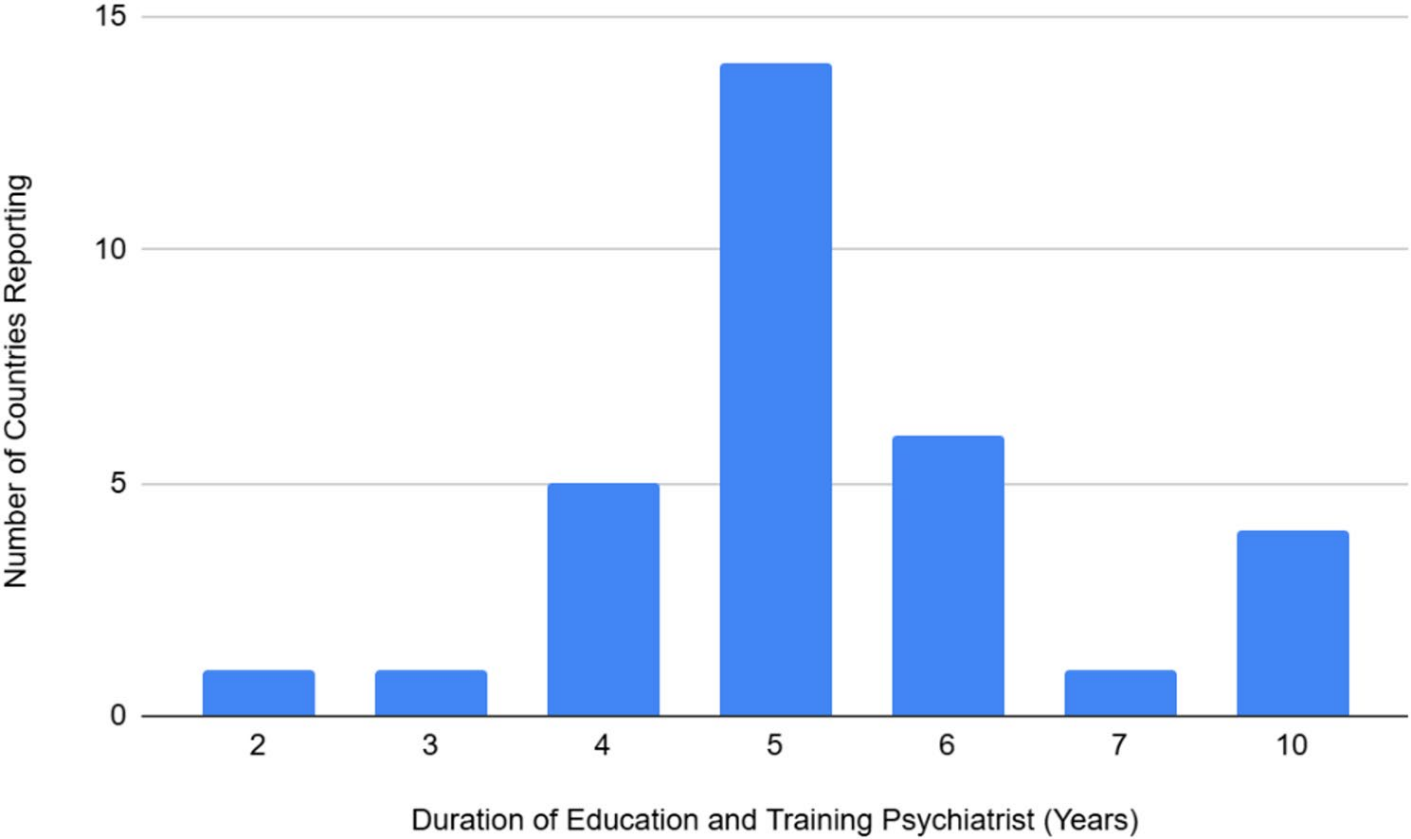
Number of countries reporting and duration of education and training psychiatrist

Recognition of qualifications was reported as a post-secondary PSM diploma (more than 4 years) followed by a general medical degree before specialization in psychiatry. There was evidence of variations in the length of training in psychiatry following a medical degree. The minimum duration was reported as 2 years in one country, and the maximum duration was reported as 10 years in four countries. Fourteen countries most reported a duration of 5 years (mean 5.17, median 5.0), with other durations including six years (six countries), four years (five countries), seven years (one country), and three years (one country) (Fig. 1).

In general, there were no differences in the length of training reported by countries between child and adolescent psychiatry specialties, general psychiatry and forensic psychiatry. A total of three countries showed variations in training. This included two years for children and adolescent training compared with three years for adult psychiatry. One country reported two years for forensic and adult psychiatry compared with three years for child and adolescent psychiatry. In contrast, one country reported five years of training for child and adolescent psychiatry compared to three years for general adult psychiatry.

### Description of activities

There were commonalities across countries in the description of activities for psychiatrists. The terms commonly used to describe activities included: bio-psychosocial, diagnosis, treatment and prevention, specialized assessment, psychotherapy, sociotherapeutic, and psychotherapeutic and psychiatric rehabilitation. These were in addition to a strategic and leadership role in developing evidence-based practice and standards of practice.

## Psychiatric and mental health nurses

### Regulatory practices and duration of education and training

A total of 12 out of the 32 countries reported the regulation of psychiatric nursing as a “protected title” and “recognized as a specialist role”. However, it is important to note that 20 countries did not report on regulation or entry-level qualification, and 19 countries did not provide information on the length of education and training for psychiatric/mental health nurse training. The main titles reported included psychiatric nurse, mental health nurse, and nurse specialized in mental health.

The recognition of qualifications varied and was reported from SEC-Certificate attesting the completion of a secondary level course (one country), DSE- Diploma from post-secondary level (two countries), PS3 Diploma post-secondary level, 3–4 years (three countries), PS4 Diploma post-secondary level, 4 years (three countries) to PSM Diplomas post-secondary level, more than 4 years (three countries) (Table 3).

**Fig. 2:**
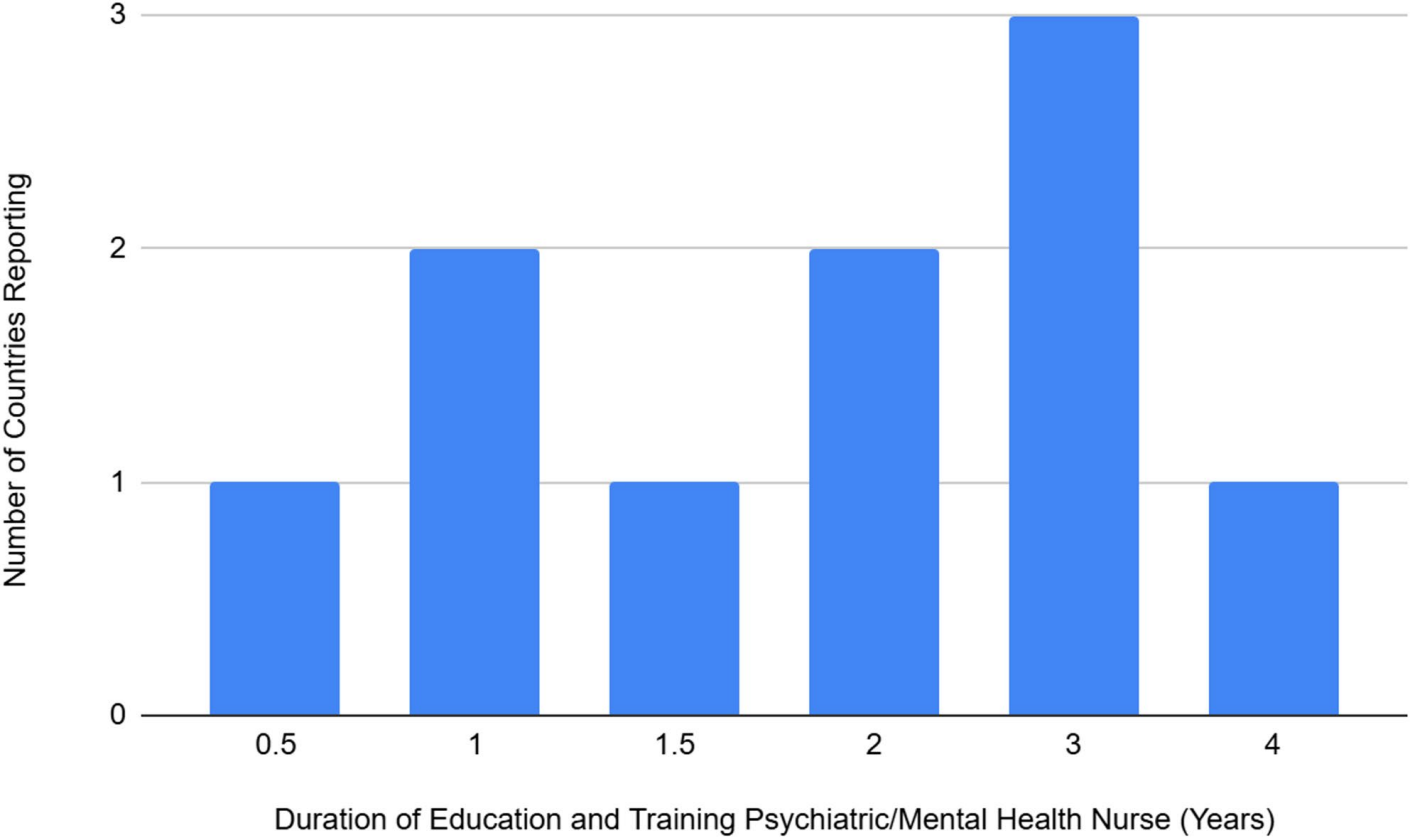
Number of countries reporting and duration of education and training in years for psychiatric and mental health nurses

Similar to psychiatrists, there were differences in the duration of education and training for psychiatric and mental health nurses. Among the countries providing data (*N* = 10) the average duration of education training was 2.0 years (median 2.0 years) with a minimum duration of 0.5 years (reported in one country) and a maximum of 4 years (reported in one country). A duration of 3 years was reported in three countries, followed by two years in two countries, 1.5 years in one country and 1 year in two countries (Fig. 2). The number of countries that require general nurse training prior to specialist nurse training is less clear, with only two countries reporting that general nursing is required for two years followed by specialist training in mental health for two years. Two countries reported a specialist psychiatric and mental health nurse training program at degree level over 4 years, followed by registration on the psychiatric division of the regulators nursing register.

### Description of activities

The description of activities described the independent role of psychiatric and mental health nurses in the assessment, diagnosis, and providing clinical expertise in managing complex mental health issues. These were mentioned in addition to providing education and training for other health care professionals. Common terms used to describe the role included expert clinical nursing, protection, promotion and optimization of health, rehabilitation and recovery, person centered care, teaching and guide, and operating as part of an interdisciplinary team.

## Psychologists

### Regulatory practice and duration of education and training

Of the 32 European countries, 29 countries regulate the role of psychologists with 10 countries reporting that the title is “protected without reserves of activities”, 9 countries reported it as a “protected title with reserves of activities”, three countries reported as “reserves of activities”, and six countries reported that the role is “regulated under legislation”. There were several titles used to describe a profession of psychologists including forensic psychologist, neuropsychologist, healthcare psychologist, clinical psychologist, clinical educational psychologist, adult clinical and mental hygiene psychologist, child and adolescent clinical and mental hygiene psychologist, and applied health psychologist and clinical orthopedagogue.

**Fig. 3:**
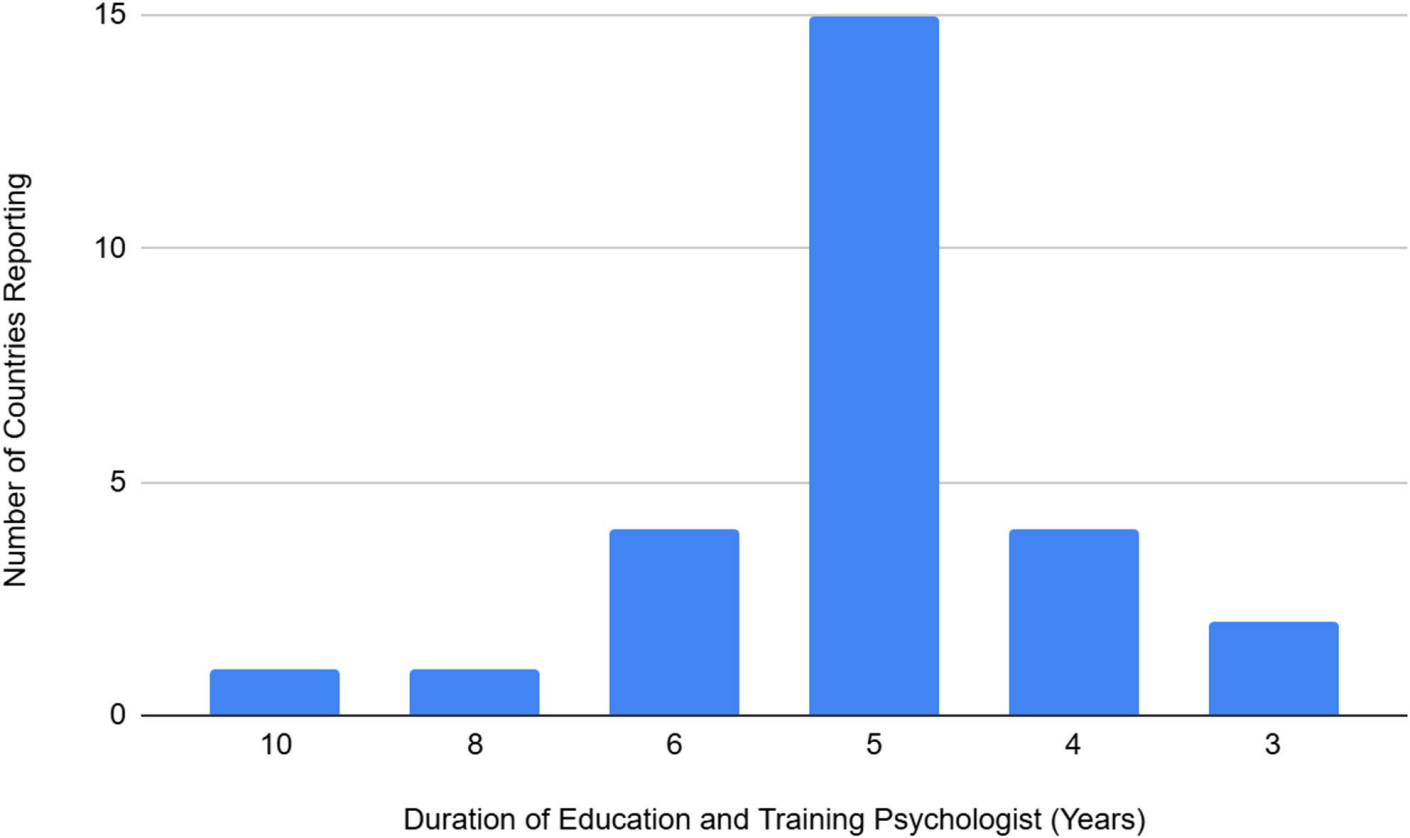
Number of countries reporting and education and training in years psychologist

Data on the duration of education and training was provided by 26 countries, with an average duration of 5.19 years (median 5.0). There was evidence of variation in the duration of education and training between countries, with a minimum of 3 years reported by two countries and a maximum of 10 years reported by one country. There were also commonalities with 15 countries reporting the duration of education and training as 5 years, followed by four countries reporting 6 years, four countries reporting 4 years, and one country reporting 8 years (Fig. 3).

The recognition of the qualification level was generally described as PSM- Diploma from post-secondary level (more than 4 years) reported by 27 countries, and Diploma from post-secondary level (more than 4 years) reported in two countries (Table 3).

### Description of activities

The terms used to describe professional activities for psychologists included assessment and examination, and detection, identifying high-risk behavior, promoting and maintaining health. Psychological diagnostics and treatments, counselling, promotive and preventive work were commonly referenced. Other common terms included psychotherapy, therapeutic interventions, rehabilitation, recovery, and teaching and training.

## Occupational therapists

### Regulatory practices and duration of education and training

Out of 32 countries, a total of twelve countries reported on the regulatory practices and duration of education and training for occupational therapists. Ten countries reported that the role of the occupational therapist is regulated with two countries reporting NIL indicating that the role is not regulated. Types of regulation included “reserves of activities with a protected title” reported by six countries, “reserves of activities” reported by four countries, and a “protected title reported without reserves of activities” reported by two countries.

**Fig. 4:**
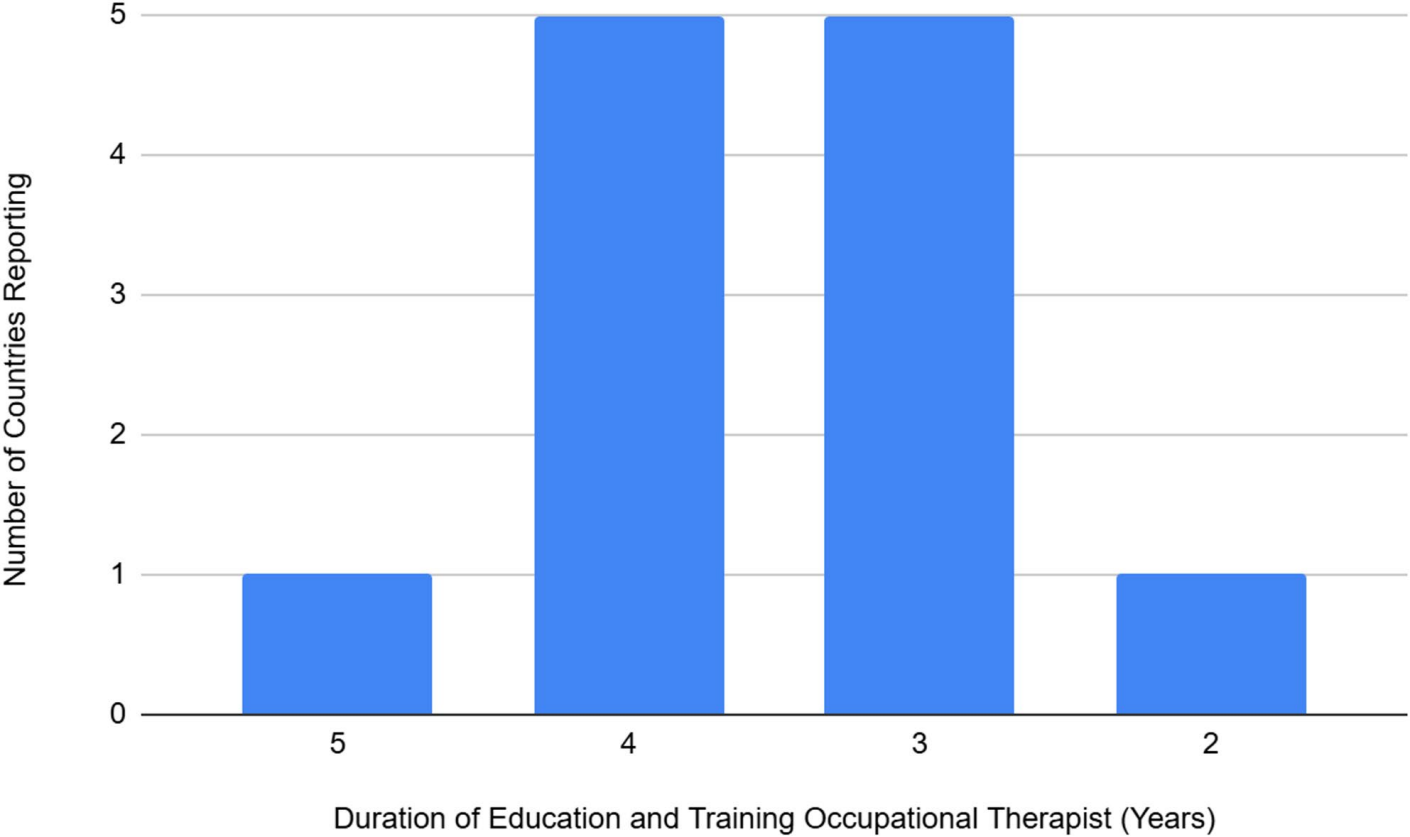
Occupational therapists average length of education and training and number of titles

The title of occupational therapist was recognized by 11 countries, with the addition of the title of a curative teacher reported by one country. The average duration of education and training was 3.5 years (median 3.5), with a minimum of 2 years reported in one country and a maximum of 5 years reported in one country. A total of five countries reported 3 years and five countries reported 5 years (Fig. 4).

The level of qualification was described as general post-secondary education and vocational post-secondary education. The range of qualification levels varies from PS3- diploma of post-secondary level (3–4 years) reported by nine countries, DSE-diploma post-secondary (4 years) reported by two countries, and PSM- diploma of post-secondary level reported by one country (Table 3).

### Description of activities

The description of the activities of the occupational therapists included some information to describe their work in mental health settings, such as therapeutic and educational assistance to individuals with development or behavioral disorders, early interventions and psychosocial rehabilitation, and treatments based on neuropsychological and psychosocial interventions. Other key terms described crisis interventions and guidance to improve the ability of people with mental health conditions to carry out everyday activities.

## Social workers

### Regulatory practices and duration of education and training

A total of nine out of 32 countries provided data on regulatory practices and education and training for social workers that referred to mental health in the description of activities. The type of regulation was mainly “reserves of activities without a protected title,” reported by six countries, followed by “reserves of activities,” reported by two countries and “reserves of activities and a protected title,” reported by one country. Several different titles were used to describe the profession of the social worker including social worker, social pedagogue counsellor, social and child welfare helper specialist, social and child welfare development specialist, social pedagogue, basic social counselling, specialist social counselling, social worker counsellor, and social pedagogue counsellor.

**Fig. 5:**
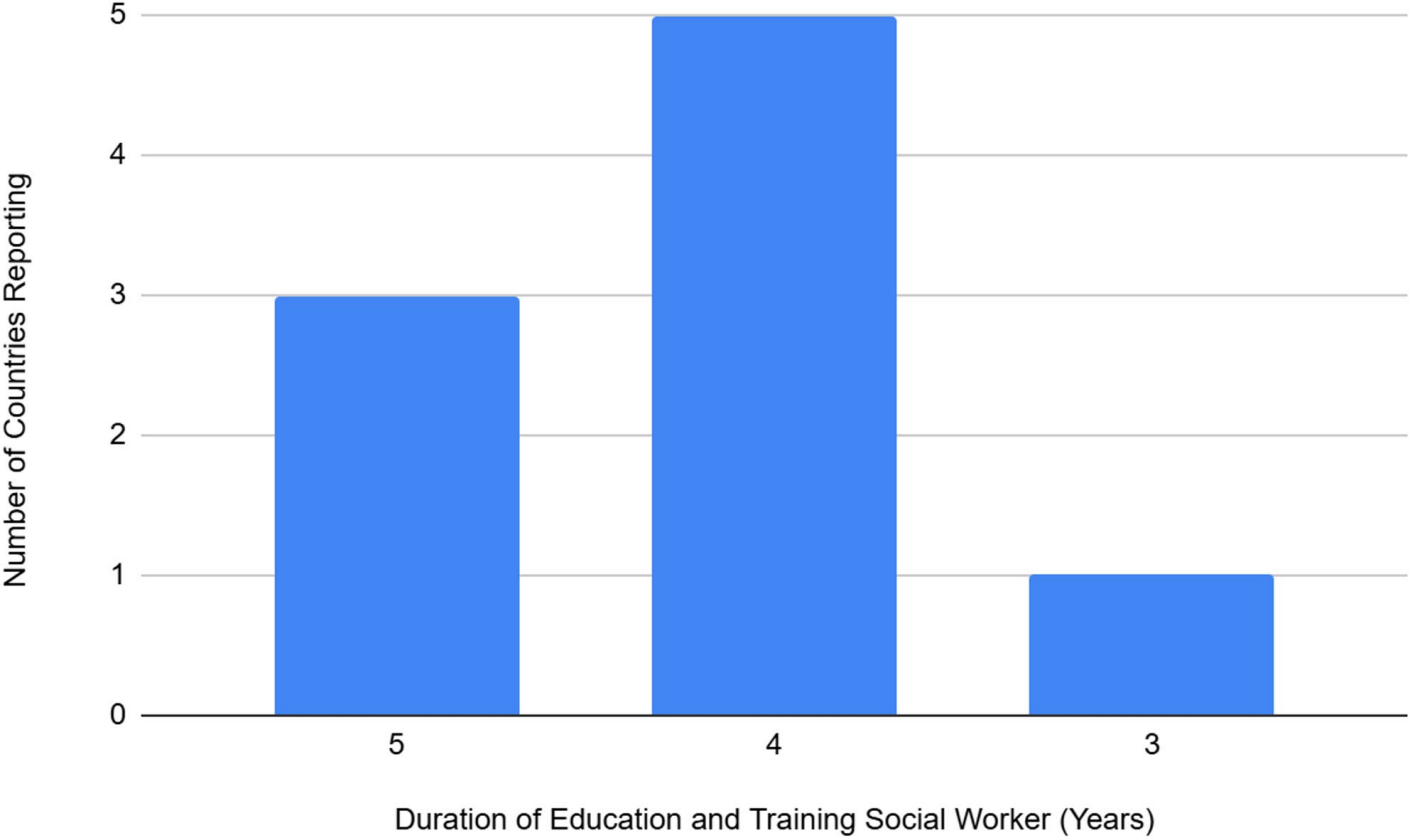
Number of countries reporting and duration of education and training

Compared to the other professions, there was less variation in the duration of education and training with an average of 4.25 years (median 4.0) and a minimum reported as 3 years by one country and maximum reported as 5 years by 3 countries, with four countries reporting 4 years duration (Fig. 5). Qualification recognitions were varied reported as PS3 diploma from post-secondary level (3–4 years) reported by four countries, PSM Diploma from post-secondary level (more than 4 years) reported by two countries, PS4 Diplomas of post-secondary level (4 years), reported by one country, DSE Diploma of post-secondary education (3–4 years) reported by one country, and SEC- certificate attesting to the completion of a secondary course reported by one country (Table 3).

### Description of activities social workers

The main descriptions of activities were similar to those of the other four professions, including prevention, detection, diagnosis, early intervention and treatment, counselling and guidance on different solutions in the care of children, adolescents and adults at risk of disorders or behavioral problems and their social environment.

## Discussion

The transformation of mental health services requires an understanding of change across multiple perspectives and at different levels of the service system [10]. From the viewpoint of the mental health workforce, the available information has mainly focused on the number of different professionals working in specialized settings, with little information from primary healthcare and community-based services [10, 15]. Transformation involves not only organizational changes and service provision in new settings but also challenges professional identities and the scope of practice of professionals. Professionals working in these settings, as well as the professions’ education, need to adapt to this change. However, it is difficult to implement change without knowledge of the current situation. Therefore, this study aims to describe the current knowledge on the five mental health professions in Europe, using information from the European Commission’s Regulated Professions Database, to provide a baseline for further studies based on the same database, as well as a knowledge base for further discussions.

### Type of regulation

The regulation viewpoint was chosen as one perspective as it provides a structure, process, and method to increase the visibility of the mental health workforce [27], although there are also critical viewpoints concerning regulation potentially seen as a way of controlling or following the ideas of a new public management approach [28] as well as professions’ attempts to maintain “social closure” or setting boundaries to exclude the profession’s non-members [29, 30]. The role of psychiatrist appears to be the most clearly regulated, as all countries reported both reserved activities and a protected title. A similar situation was observed for psychiatric and mental health nurses; however, the number of reporting countries was low, with only 12 out of 32 providing data, despite nurses being the largest professional group in the mental health field [31]. This observation may reflect the problematic area of education to achieve the competence of psychiatric and mental health nurses, which is not clearly defined at European level, as well as the lack of regulatory practice in many European countries. Similarly, there was no data available on advanced practice level mental health nurses although several countries in Europe have been developing the advanced nursing role and education for many years [32]. Unlike other professions, only two countries reported regulation under national legislation. Further country-based analysis would be required to identify the regulatory practices in these countries. For social workers and occupational therapists, the regulations varied from the protection of titles to the reservation of activities; however, the number of reporting countries was low.

While recognizing differences between European countries is important, some of these knowledge gaps could be addressed through alternative regulatory approaches aimed at improving quality and safety standards in health and social care systems [33]. European standardization of regulatory practices offers one solution to ensuring the competence of mental health professionals working with vulnerable populations across all service levels [19, 27].

### Duration of education and training

The results on the duration of education and training show a great deal of variation. For example, for psychiatrists, with a minimum of 2 years and a maximum of 10 years of training were reported. When compared with the EU Directive [21], which sets a minimum training period of 4 years, the results suggest that there are differences in some countries with fewer years, while others might have also included the previous medical education incorporated in the 10-year time frame. Although the EU Directive applies to general nurses, it does not specifically address psychiatric and mental health nurses, a group that also exhibited considerable variations in training length, ranging from 0.5 to 4 years. This may also include previous education as a nurse in general care.

The absence of occupational therapists, social workers, and psychologists from the EU Directive likely contributes to the significant variations observed in their education and training. For example, the reported minimum training duration for psychologists was 3 years, while the maximum reported was 10 years. Similarly, the length of education for occupational therapists varied from 2 to 5 years. In contrast, the training duration for social workers appeared more consistent across the reporting countries.

### Descriptions of activities

From the perspective of service transformation, the declaration of activities is interesting in understanding how these are translated into practice and the definition of competence required in different mental health settings. The study revealed two significant patterns in the description of activities. First, non-medical professions’ activities were sometimes described using medical terms, which, in the case of psychiatric and mental health nurses, may suggest a lack of independent professional recognition across Europe, potentially casting them in a medical or support role in some countries. At the same time some countries reported on the independent role of the psychiatric and mental health nurse. Second, the descriptions predominantly focused on individual interventions, with limited mention of the crucial role of social networks and families in community-based care.

While psychiatrists and psychiatric and mental health nurses predominantly work in specialized services, social workers, occupational therapists, and psychologists are employed across a wider range of service areas beyond mental health. This might explain the variations in their description of activities. Overall, without standardization, it is difficult to determine the precise role of each profession as shown also with these results on description of activities of this study. At the same time this lack of clear boundaries may reflect the multidisciplinary nature of mental healthcare today [34].

This descriptive study has revealed gaps in knowledge and differences in reporting and regulatory practices between European countries in the five mental health professions. This underlines the need for further research to improve understanding of professional competence equivalence and the specific development required to drive effective transformation of mental health services. This study also provides a baseline of information with which to track the transformation of mental health services from a workforce perspective, using data from the Regulated Professions Database.

### Limitations of the study

While the European Commission’s database on regulated professions serves the purpose of supporting mobility, it does not offer the in-depth information required for a comprehensive understanding of the professions studied. The reliability of the information included in the database depends on national reporting at country level - and on the availability of national information. This became evident also in this study; for example, information on the duration of education was often lacking and in some countries the length of education indicates that the different levels of education are combined, such as bachelor and master education. The results also show variations in the descriptions of activities, which might reflect cultural and terminological differences between countries. In general, the number of reporting countries was low for some professions. The heterogeneity and the extent of missing data limited the statistical possibilities for comparative or inferential analysis. During the data screening phase, manual extraction of the professions of psychiatrists and psychiatric and mental health nurses from among all medical doctors and nurses posed a risk of non-recognition, which was mitigated through collaboration between two researchers.

Therefore, the results of this study should be considered as preliminary and descriptive, requiring further research. At the same time the knowledge gaps raise concerns about mental health policy implementation and transformation without regulations ensuring reliable data on workforce capacity and competence for safe and effective mental health care. As the study focused on five mental health professions, country-specific mental healthcare professions, peer support workers and family members were excluded, despite their important role in mental health care. Therefore, the results of this descriptive study do not provide a comprehensive picture of the mental health care workforce. With these limitations in mind, some general conclusions can be drawn from the results.

## Conclusions

Drawing inspiration from Liljegren & Saks [35] on the use of metaphors for professions, this study’s findings suggest viewing the mental health workforce landscape as an image where psychiatrists form a distinct, sharply defined island within the broader sea of mental health care. In contrast, other professions present a more blurred and indistinct picture. To achieve a truly comprehensive understanding of this workforce, all professions, including unqualified staff, those with diverse titles, peer support workers, and family members, need to be as clearly delineated as psychiatrists, recognizing their integral resources and roles as change agents in the ongoing transformation. These views can be strengthened by adopting different ways to regulate and improve quality and safety standards in health and social care systems [33]. European standardization of regulatory practices offers one solution [19, 27] to ensure the competence of mental health professionals working with vulnerable populations across all service levels. As the service system changes, regulation, competence and capability requirements and professional scopes of practice need to be revisited to provide high-quality mental health care in the new environments.

## Data Availability

Not applicable.
